# miRNA-200b—A Potential Biomarker Identified in a Porcine Model of Cardiogenic Shock and Mechanical Unloading

**DOI:** 10.3389/fcvm.2022.881067

**Published:** 2022-05-25

**Authors:** Christian Riehle, Jan-Thorben Sieweke, Sayan Bakshi, Chae-Myeong Ha, Nanna Louise Junker Udesen, Ole K. Møller-Helgestad, Natali Froese, Hanne Berg Ravn, Heike Bähre, Robert Geffers, Roland Seifert, Jacob E. Møller, Adam R. Wende, Johann Bauersachs, Andreas Schäfer

**Affiliations:** ^1^Department of Cardiology and Angiology, Hannover Medical School, Hanover, Germany; ^2^Division of Molecular and Cellular Pathology, Department of Pathology, University of Alabama at Birmingham, Birmingham, AL, United States; ^3^Department of Cardiology, Cardiothoracic Surgery and Intensive Care, Odense University Hospital, Odense, Denmark; ^4^Department of Cardiothoracic Anesthesia and Intensive Care, Rigshospitalet, Copenhagen, Denmark; ^5^Research Core Unit Metabolomics, Hannover Medical School, Institute of Pharmacology, Hanover, Germany; ^6^Helmholtz Centre for Infection Research, Research Group Genome Analytics, Braunschweig, Germany

**Keywords:** cardiogenic shock, circulating biomarkers, mechanical unloading, metabolomics, RNA sequencing

## Abstract

**Background:**

Cardiogenic shock (CS) alters whole body metabolism and circulating biomarkers serve as prognostic markers in CS patients. Percutaneous ventricular assist devices (pVADs) unload the left ventricle by actively ejecting blood into the aorta. The goal of the present study was to identify alterations in circulating metabolites and transcripts in a large animal model that might serve as potential prognostic biomarkers in acute CS and additional left ventricular unloading by Impella ^®^ pVAD support.

**Methods:**

CS was induced in a preclinical large animal model by injecting microspheres into the left coronary artery system in six pigs. After the induction of CS, mechanical pVAD support was implemented for 30 min total. Serum samples were collected under basal conditions, after the onset of CS, and following additional pVAD unloading. Circulating metabolites were determined by metabolomic analysis, circulating RNA entities by RNA sequencing.

**Results:**

CS and additional pVAD support alter the abundance of circulating metabolites involved in Aminoacyl-tRNA biosynthesis and amino acid metabolism. RNA sequencing revealed decreased abundance of the hypoxia sensitive miRNA-200b following the induction of CS, which was reversed following pVAD support.

**Conclusion:**

The hypoxamir miRNA-200b is a potential circulating marker that is repressed in CS and is restored following pVAD support. The early transcriptional response with increased miRNA-200b expression following only 30 min of pVAD support suggests that mechanical unloading alters whole body metabolism. Future studies are required to delineate the impact of serum miRNA-200b levels as a prognostic marker in patients with acute CS and pVAD unloading.

## Introduction

Acute myocardial infarction complicated by cardiogenic shock (AMI-CS) is a life threatening condition with a mortality rate greater than 50% (1). AMI-CS is characterized by decreased cardiac output, organ hypoperfusion, tissue hypoxia, and cellular damage, which may result in multi-organ failure and death. Several therapeutic approaches have been developed to increase cardiac output and preserve organ perfusion in AMI-CS patients, including extracorporeal membrane oxygenation (ECMO), intra-aortic balloon pump (IABP) counterpulsation, and catheter-based percutaneous ventricular assist device (pVAD) support. While mechanical circulatory support is an intuitive therapeutic approach, IABP counterpulsation did not reduce 30-day mortality in AMI-CS patients (2), and is therefore not recommended by current guidelines (3). The Impella ^®^ pVAD (Abiomed, Danvers, MA, United States) is a promising approach used to actively unload the left ventricle (LV) by ejecting blood across the aortic valve into the ascending aorta (4–7). Ongoing studies investigate whether Impella ^®^ pVAD support improves survival in patients with AMI-CS (8). Therefore, it is of great interest to identify circulating biomarkers under conditions of AMI-CS and following pVAD unloading, which can easily be determined in patients on a routine basis.

Circulating metabolites are biomarkers for several diseases and are used in predictive risk scores of cardiogenic shock (CS) (9). Metabolites reflect alterations in organ perfusion and indicate cellular damage. Similar to metabolites, non-coding RNAs (ncRNA), including microRNAs (miRNAs), have a great potential to serve as biomarkers for numerous diseases, including cardiomyopathies (10, 11). ncRNAs can be detected in tissues and in fluids and are master regulators of metabolism (12, 13). Therefore, serum levels of ncRNAs provide a molecular fingerprint and are a powerful tool to assess changes in whole body metabolism.

The goal of the present study was to identify signatures in circulating metabolites and transcripts in AMI-CS and following LV mechanical unloading by pVAD, which might serve as potential biomarkers. We therefore used a large animal model and performed a multi-omics approach to identify changes in the serum metabolome and circulating transcripts.

## Materials and Methods

### Animal Studies

**FIGURE 1:**
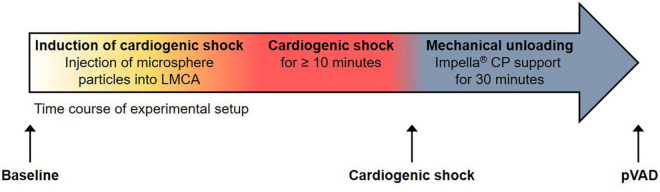
Experimental setup. Time course of experimental setup with induction of cardiogenic shock and percutaneous ventricular assist device (pVAD) support by Impella ^®^ CP. Serum samples were collected at the time points denoted by black arrows. Note that the time point “pVAD” refers to pVAD support in the presence of cardiogenic shock.

Animal experiments were conducted with approval from and in accordance with guidelines from the Danish Animal Experiments Inspectorate at the University of Southern Denmark, Odense, Denmark (authorization number: 2016-15-00951). The investigation conforms to the Guide for the Care and Use of Laboratory Animals published by the US National Institutes of Health (NIH Publication No. 85-23, revised 1985). Six Danish Landrace female pigs were studied (14). Animals were selected based on the availability of serum samples for further analysis. The individual hemodynamic response to the induction of CS and mechanical unloading was not considered for the selection of serum samples. The experimental protocol for anesthesia, the induction of CS, and mechanical unloading by Impella ^®^ CP pVAD support has been described in detail (14). Briefly, after induction of anesthesia, CS was induced by stepwise injecting 0.125 g polyvinyl alcohol microsphere particles (Contour™; Boston Scientific, Marlborough, MA, United States) mixed with 10 ml saline and 10 ml contrast agent into the left main coronary artery (LMCA) (15). Hemodynamics were allowed to stabilize for the duration of 2–3 min after each injection prior to the administration of the next 1 ml injection. CS was defined as a reduction of SvO_2_ to < 50% of the Baseline value or an absolute value of < 30% and/or cardiac index < 1.5 l/min/m^2^ sustained for the duration of at least 10 min. Mechanical support with an Impella ^®^ CP pVAD was initiated after the onset of CS and was continued for the remaining duration of the study of 30 min. Impella ^®^ CP support was initiated at full speed (performance level 8), which resulted in an average flow rate of 3.18 ± 0.10 l/min. Impella performance was similar across all animals investigated. After completion of the studies, animals were sacrificed by a lethal dose of pentobarbital (Figure 1 and Supplementary Table 1).

### Serum Collection

Venous blood samples were collected at the time points indicated in Figure 1 and were immediately centrifuged at 2,000 g for 10 min at 4^°^C. The liquid supernatant was snap frozen in liquid nitrogen and stored at −80°C until subjected to further analysis.

### RNA Sequencing Analysis

RNA from serum samples was isolated using the miRNeasy Serum/Plasma Advanced Kit (Qiagen, Hilden, Germany) according to the manufacturer’s instructions. Quality and integrity of total RNA was controlled using a 2100 Bioanalyzer System (Agilent Technologies, Waldbronn, Germany). RNA sequencing and bioinformatic analysis were performed as described in detail in Supplementary Material. RNA sequencing data have been deposited to the GEO database under the accession number GSE199090.

### Metabolomic Analysis

To detect whole body metabolic alterations in CS and additional pVAD support, a LC-MS and GC-MS metabolomic analysis using serum samples was performed as described in detail in the Supplementary Material.

### Statistical Analysis

See the Supplementary Material for details.

## Results

### Metabolomic Profiling of Serum

**FIGURE 2:**
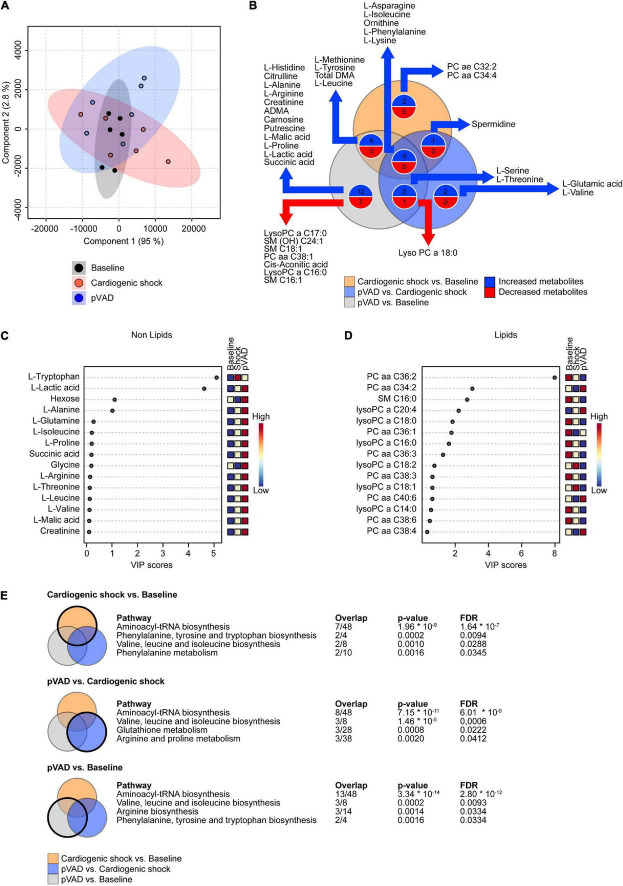
Metabolomic profiling of serum. **(A)** Partial Least Squares-Discriminant Analysis (PLS-DA) visualizing global metabolite abundance. **(B)** Venn diagram illustrating the number of altered metabolites as determined by LC-MS and GC-MS metabolomic analysis. Numbers of metabolites presented in the Venn diagram correspond to *p* < 0.05. **(C,D)** Variable importance in projection (VIP) score analyses. **(E)** Significantly enriched pathways for the comparisons as indicated (cut-off: false discovery rate (FDR) *p* < 0.05); pVAD, percutaneous ventricular assist device.

A total of 151 metabolites was analyzed (Figures 2A,B, Supplementary Figure 1, and Supplementary Table 2). We observed an increased abundance of 12 metabolites following the induction of CS compared to Baseline (L-Methionine, L-Tyrosine, Total DMA, L-Leucine, L-Asparagine, L-Isoleucine, Ornithine, L-Phenylalanine, L-Lysine, PC ae C32:2, PC aa C34:4, and Spermidine). pVAD support during CS increased the abundance of 10 metabolites (L-Serine, L-Threonine, L-Asparagine, L-Isoleucine, Ornithine, L-Phenylalanine, L-Lysine, Spermidine, L-Glutamic acid, and L-Valine). LysoPC a C18:0 was the only metabolite that decreased following pVAD support during CS. A total of 23 metabolites was increased and 8 metabolites were decreased in the comparison pVAD relative to Baseline (Figure 2B). To identify biomarkers that are regulated by pVAD unloading in CS, we focused on metabolites that were altered in the comparisons CS vs. Baseline and pVAD vs. CS in the opposite direction. No metabolite and pathway were identified by our bioinformatics analysis after applying these selection criteria. Variable importance in projection (VIP) score analyses were separately performed for lipid species and the remaining metabolites. This analysis identified L-Tryptophan, L-lactic acid and hexose as the top three characteristic non-lipids (Figure 2C), and Phosphatidylcholine diacyl C 36:2 (PC aa C36:2), Phosphatidylcholine diacyl C 34:2 (PC aa C34:2) and Shingomyeline C 16:0 (SM C16:0) as the top three characteristic lipids (Figure 2D) to separate groups. Our pathway analysis identified Aminoacyl-tRNA biosynthesis and valine, leucine, and isoleucine biosynthesis as the top regulated pathways for the comparisons CS vs. Baseline, pVAD vs. CS, and pVAD vs. Baseline (Figure 2E). Together, these data indicate that the identified circulating metabolites (summarized in Supplementary Table 2) do not serve as biomarkers in AMI-CS and pVAD-mediated unloading since no metabolite was identified that was altered in the comparisons CS vs. Baseline and pVAD vs. CS in the opposite direction.

### Transcriptomic Profiling of Serum

**FIGURE 3:**
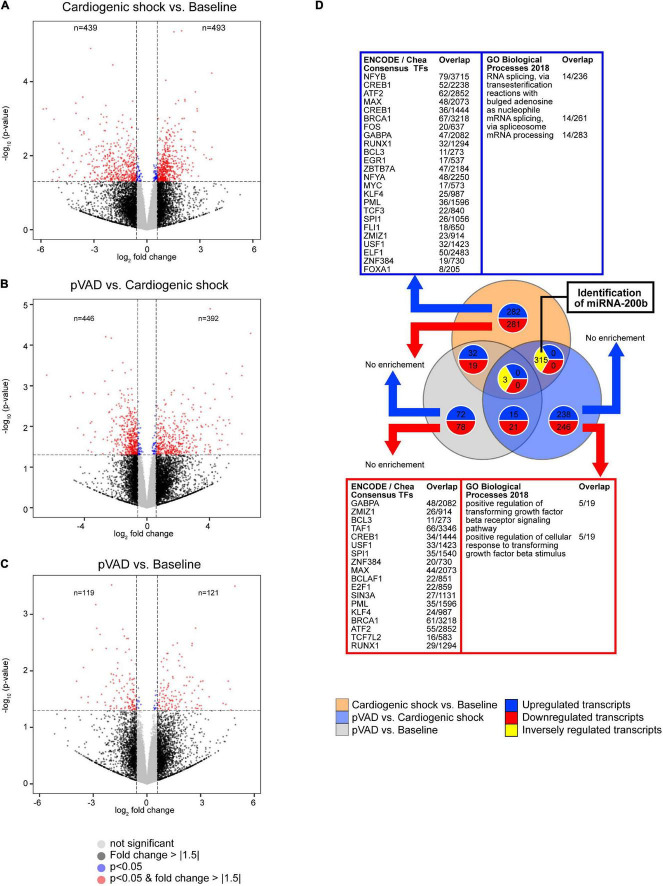
Transcriptional profiling determined by RNA sequencing. Volcano plots for the comparisons **(A)** Cardiogenic shock vs. Baseline, **(B)** pVAD vs. Cardiogenic shock, and **(C)** pVAD vs. Baseline. Volcano plots display the expression level of all transcripts detected. Dashed lines indicate *p* = 0.05 (equals -log_10_ ∼ 1.30) and a | fold change| > 1.5 (equals an absolute log_2_ fold change ∼ 0.585). Numbers indicate the number of different expressed transcripts with a | fold change| > 1.5 and *p* < 0.05. **(D)** Venn diagram illustrating the number of altered transcripts for the RNA sequencing experiments performed. The number of transcripts presented in the Venn diagram correspond to a | fold change| > 1.5 and *p* < 0.05. Significantly enriched transcription factors and pathways as indicated (*q* < 0.1); pVAD, percutaneous ventricular assist device.

To detect transcripts that are altered in CS and following pVAD-mediated unloading that might serve as potential prognostic biomarkers, we performed a RNA sequencing experiment using aliquots of the same serum samples that were subjected to our metabolomic analysis. We detected a total of 26,146 transcripts; 932 of these transcripts were altered for the comparison CS vs. Baseline (*n* = 493 increased, *n* = 439 decreased), 838 transcripts for the comparison pVAD vs. CS (*n* = 392 increased, *n* = 446 decreased), and 240 transcripts for the comparison pVAD vs. Baseline (*n* = 121 increased, *n* = 119 decreased; cutoff each: *p* < 0.05 and a | fold change| > 1.5; Figures 3A–C, Supplementary Figures 2, 3, and Supplementary Tables 3, 4). Our bioinformatic pathway enrichment analysis identified that increased circulating transcripts are mainly associated with RNA splicing and processing in CS relative to Baseline (cutoff: *q* < 0.1; Figure 3D). Significantly upregulated transcripts were found to be enriched for the multiple transcriptional regulators, top three being nuclear transcription factor Y subunit beta (NFYB), cAMP responsive element binding protein 1 (CREB1) and activating transcription factor 2 (ATF2). Significantly downregulated transcripts for the comparison CS relative to Baseline were associated with transforming growth factor beta signaling. GA binding protein transcription factor subunit alpha (GABPA), zinc finger MIZ-type containing 1 (ZMIZ1) and BCL3 transcription coactivator (BCL3) were identified to be the top three enriched transcriptional regulators. Similar to our metabolomics experiment and to identify potential protective pathways and transcripts that are mediated by pVAD unloading in CS, we focused on transcripts that were altered in the comparisons CS vs. Baseline and pVAD vs. CS in the opposite direction. This analysis revealed 315 transcripts and identified the hypoxia sensitive miRNA-200b, which was the only miRNA after applying these selection criteria (Figure 3D). Subsequent bioinformatic pathway analysis failed to identify a significant and specific biological pathway or upstream transcription factor regulator for this set of transcripts.

**FIGURE 4:**
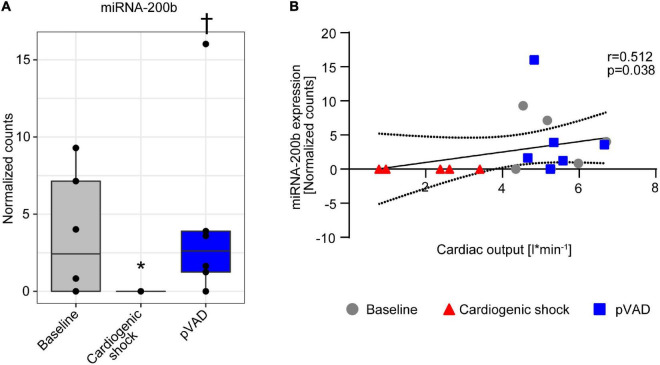
miRNA-200b expression in serum. **(A)** miRNA-200b expression at time points as indicated. Data are presented as median ± interquartile range (IQR), *n* = 5–6. *p < 0.05 vs. Baseline; ^†^*p* < 0.05 vs. Cardiogenic shock; pVAD, percutaneous ventricular assist device. **(B)** Spearman correlation with 95% confidence intervals calculated by linear regression model between miRNA-200b expression and cardiac output.

miRNA-200b expression decreased following induction of CS, which was reversed following pVAD support (Figure 4A). We next correlated miRNA-200b expression with cardiac output, which similarly completely recovered following pVAD support (Supplementary Table 1). The Spearman correlation analysis shows that miRNA-200b expression and cardiac output are positively correlated (*r* = 0.512, *p* = 0.038, Figure 4B). Together, these data indicate that pVAD support reverses the CS-mediated repression in miRNA-200b expression, which is positively correlated with cardiac output and is indicative of hypoxia-associated transcript expression.

## Discussion

The goal of the present study was to identify circulating metabolites and transcripts that are altered in one direction in CS and in the reversed direction following LV unloading by pVAD support in CS and might serve as potential biomarkers. Altered serum concentrations of amino acids have been reported for patients with septic shock and have been suggested as potential predictors of sepsis progression (16). Plasma metabolomic profiling of septic shock patients suggested that lipidome alterations might be critical for patients’ responses to infection (17).

miRNAs are ncRNAs with 19–25 nucleotides in length. They are powerful regulators of gene expression at the post-transcriptional level and serve as biomarkers for numerous disease conditions (11). miRNA-200b expression is mediated by hypoxia and is therefore named a hypoxamir (18, 19). Numerous studies reported modulation of miRNA-200b expression in response to hypoxia. While some studies reported an induction in response to hypoxia and following ischemic preconditioning (20–22), others reported impaired expression under hypoxic conditions (23). The differences might be attributable to the intensity and duration of the stimulus, the specific cell type, and the tissue investigated. Of note, pVAD support during CS completely reversed the potentially hypoxia-mediated suppression of miRNA-200b (Figure 4). This suggests a potential protective effect of pVAD support on tissue and organ perfusion in CS, which is supported by our hemodynamic data (Supplementary Table 1) and our previous studies showing increased carotid and renal blood flow following pVAD support during CS (24). Together, our studies identify miRNA-200b as a potential marker for organ hypoperfusion and ischemia in CS. Future studies are required to delineate the impact of serum miRNA-200b levels as a prognostic marker in patients with CS and pVAD unloading.

Interestingly, the reversal in the hypoxamir miRNA-200b expression following pVAD support was not paralleled by a reversal of pattern of circulating metabolites. These data suggest that mechanical unloading improves oxygen availability and might alter whole body metabolism; however, was not reflected by changes in circulating metabolites in the present study. In this context, it is critical to note that the duration of mechanical unloading was only 30 min total since we aimed to determine immediate changes in circulating transcripts and metabolites in the acute phase of CS. It will be of interest to determine whether the early change in the hypoxia-mediated transcriptional response results in changes in circulating metabolites and whole-body metabolism over a longer time period. This is the context of future studies. An additional aspect to consider is that AMI-CS patients are typically subjected to percutaneous coronary intervention to restore coronary blood flow. One could hypothesize that a “point of no return” for the recovery in LV contractile function following pVAD-support after prolonged duration of AMI-CS despite successful revascularization exists. It is therefore of interest to measure miRNA-200b expression and other markers for metabolic pathways in this setting. In clinical practice, pVAD support is used in AMI-CS patients that are subjected to percutaneous coronary intervention to restore coronary blood flow. This contrasts with the present study, in which CS was induced by injecting microspheres into the left coronary artery system that resulted in permanent vessel occlusion. Therefore, it is of interest to investigate the impact of pVAD support on circulating transcripts and metabolites in an AMI-CS model, in which coronary blood flow is temporarily impeded to mimic myocardial infarction and subsequently restored to mimic percutaneous coronary intervention. This experimental setup mimics aspects of revascularization following percutaneous coronary intervention and is of interest for the design of future studies.

Limitations of the study include that metabolites and transcripts were detected in serum, but not in different tissues. Therefore, it is impossible to discern from which organ the detected metabolites and transcripts primarily originate from. It is of great interest to correlate the changes in metabolites and transcripts in serum with tissue levels, which will be the subject of future studies.

Furthermore, mRNA is typically primarily detected in the cytoplasm and not in serum. Thus, most mRNA transcripts in the RNA sequencing analysis using serum likely emanates from degrading cells of unknown origin. Furthermore, our analysis detected a very limited number of small RNA transcripts. One explanation is that the analysis was confounded by the large number of small RNA fragments that was detected in the analysis. Most of these fragments most likely results from degraded longer RNA entities. Our bioinformatic analysis of both the metabolomics and the RNA sequencing experiment revealed a relatively great variability between samples compared to the effect of treatment (Figure 2A and Supplementary Figures 2A, 3). Importantly, data presented from our metabolomics and RNA sequencing analysis are not adjusted for multi-testing unless otherwise indicated. No significantly altered transcript was detected after adjusting for multi-testing using the Benjamini-Hochberg procedure (Supplementary Tables 3, 4). This limitation is attributable to the limitations outlined above and the small sample size of the present study. However, it is important to note that the present study is a hypothesis-generating pilot study to identify metabolites and transcripts that are altered in acute CS and following LV unloading by pVAD. The currently performed randomized multi-center DanGer shock study will determine whether mechanical circulatory support with Impella ^®^ CP improves survival in AMI-CS (8). It will be of great interest to determine whether the changes in the signature of circulating metabolites and miRNA-200b will be also observed in this patient cohort and whether they might serve as prognostic markers. This is the subject of future studies.

Another limitation is that LV unloading by pVAD was performed for the duration of only 30 min total. This contrasts with the duration of LV unloading in patients with CS, which typically lasts for several days until recovery of contractile function occurs. It is of great interest to determine whether the changes in circulating metabolites and transcripts observed in the present study will be also observed in patients. This important question and the prognostic impact of specific metabolites and transcripts will be determined in the DanGer shock trial.

## Summary and Conclusion

The present study identifies miRNA-200b as a circulating marker that is repressed in CS and is restored following pVAD support. The induction of the early transcriptional response with the induction of the hypoxamir miRNA-200b expression following only 30 min of pVAD support strongly suggests that mechanical unloading alters whole body metabolism. Longer observation periods are required to determine whether this is associated with the reversal of circulating metabolite patterns. Future studies are required to delineate the impact of serum miRNA-200b levels as a prognostic marker in patients with CS and pVAD unloading.

## Data Availability Statement

The datasets presented in this study can be found in online repositories. The names of the repository/repositories and accession number(s) can be found below: GEO, GSE199090.

## Ethics Statement

The animal study was reviewed and approved by the University of Southern Denmark, Odense, Denmark; authorization number: 2016-15-00951.

## Author Contributions

CR, J-TS, HBe, AW, JM, and AS conceived and designed the study. HBä, NF, NJ, OM-H, and JM performed experiments. CR, SB, C-MH, HBä, and RG analyzed data. CR, SB, C-MH, and RG performed statistical analyses. CR wrote the manuscript. CR, SB, and C-MH contributed to visualization. CR, RG, RS, AW, JB, and AS supervised and interpreted the results. CR, JM, and AS acquired funding. All authors critically reviewed the manuscript.

## Conflict of Interest

NJ, JM, JB, and AS received research support from Abiomed. CR received travel support from Abiomed. The funder did not have any influence on the design of the study or the data collection, analysis, and interpretation of data or the content of the manuscript. The remaining authors declare that the research was conducted in the absence of any commercial or financial relationships that could be construed as a potential conflict of interest.

## Publisher’s Note

All claims expressed in this article are solely those of the authors and do not necessarily represent those of their affiliated organizations, or those of the publisher, the editors and the reviewers. Any product that may be evaluated in this article, or claim that may be made by its manufacturer, is not guaranteed or endorsed by the publisher.

## Supplementary Material

Supplementary Table 2 Metabolomics.

Supplementary Table 3 All transcripts.

Supplementary Table 4 Differentially expressed transcripts.
