# Supplementary material for: miRNA-200b—A Potential Biomarker Identified in a Porcine Model of Cardiogenic Shock and Mechanical Unloading
Source: Front Cardiovasc Med. 2022 May 25;9:881067. doi: 10.3389/fcvm.2022.881067 (PMC9174458; doi:10.3389/fcvm.2022.881067)
Supplement: Supplementary file 1 [file Data_Sheet_1.PDF]

SUPPLEMENTARY MATERIAL

FOR

miRNA-200b - A potential biomarker identified in a porcine model of cardiogenic shock and mechanical unloading

Christian Riehle[1,†,*], Jan-Thorben Sieweke[1,†], Sayan Bakshi[2,†], Chae-Myeong Ha[2,†], Nanna Louise Junker Udesen[3], Ole K. Møller-Helgestad[3], Natali Froese[1], Hanne Berg Ravn[4], Heike Bähre[5], Robert Geffers[6], Roland Seifert[5], Jacob E. Møller[3], Adam R. Wende[2], Johann Bauersachs[1], and Andreas Schäfer[1]

[1] Department of Cardiology and Angiology, Hannover Medical School, Hannover, Germany
[2] Division of Molecular and Cellular Pathology, Department of Pathology, University of Alabama at Birmingham, Birmingham, AL 35294, USA
[3] Department of Cardiology, Cardiothoracic Surgery and Intensive Care, Odense University Hospital, Odense, Denmark
[4] Department of Cardiothoracic Anesthesia and Intensive Care, Rigshospitalet, Copenhagen, Denmark
[5] Research Core Unit Metabolomics, Institute of Pharmacology, Hannover Medical School, Hannover, Germany
[6] Helmholtz Centre for Infection Research, Research Group Genome Analytics, Braunschweig, Germany

[†] These authors have contributed equally to this work and share first authorship.

**Parameter**	**Baseline**	**Cardiogenic shock**	**pVAD**
Stroke work [mmHg*mL]	5055 ± 512	789 ± 233	1099 ± 221
Potential energy [mmHg*mL]	4927 ± 798	5302 ± 911	3078 ± 359
Pressure volume area [mmHg*mL]	9982 ± 897	6090 ± 1065	4177 ± 477
Heart rate [min^{-1}]	80.3 ± 4.1	78.5 ± 2.9	70.8 ± 2.6
Left ventricular work 10^3 * [mmHg*mL] / min	810 ± 92	487 ± 97	295 ± 35
Left ventricular elastance [mmHg/mL]	1.17 ± 0.13	0.34 ± 0.02	0.62 ± 0.08
LVEDV [mL]	171 ± 11	211 ± 17	143 ± 8
LVEDP [mmHg]	15.9 ± 1.5	22.3 ± 1.8	17.3 ± 1.8
LVESV [mL]	103.8 ± 11.2	183.8 ± 14.3	111.7 ± 7.4
LVESP [mmHg]	103.2 ± 8.2	59.2 ± 5.9	60.8 ± 6.0
Mean arterial pressure [mmHg]	78.8 ± 4.6	42.0 ± 3.8	58.5 ± 4.4
Mean pulmonary arterial pressure [mmHg]	21.5 ± 1.1	24.8 ± 1.8	23.2 ± 2.1
Arterial lactate [mmol/L]	1.5 ± 0.3	1.8 ± 0.3	2.0 ± 0.4
Mixed venous oxygen saturation [%]	73.9 ± 4.4	35.2 ± 4.1	48.8 ± 3.9
Renal venous oxygen saturation [%]	72.9 ± 14.6	46.1 ± 11.4	65.2 ± 14.1
pVAD support [L/min]			3.18 ± 0.10
Cardiac output [L/min]	5.33 ± 0.36	2.10 ± 0.42	5.38 ± 0.29

Supplementary Table 1: *Hemodynamic characteristics of animals investigated at Baseline, Cardiogenic shock, and additional pVAD support.*

Data are presented as Mean ± SEM. LVEDP, left ventricular end-diastolic pressure; LVEDV, left ventricular end-diastolic volume; LVESP, left ventricular end-systolic pressure; LVESV, left ventricular end-systolic volume. Left ventricular work equals heart rate * pressure volume area. Note that data presented in this table (n=6/group) represent a subgroup of a previously published dataset (1). pVAD, percutaneous ventricular assist device. The following formula was used to calculate cardiac output: (LVEDV - LVESV) * heart rate. pVAD support was performed at full speed (performance level 8). Cardiac output for the pVAD time point was determined using the calculation outlined above, which was added to the flow rate of pVAD support.

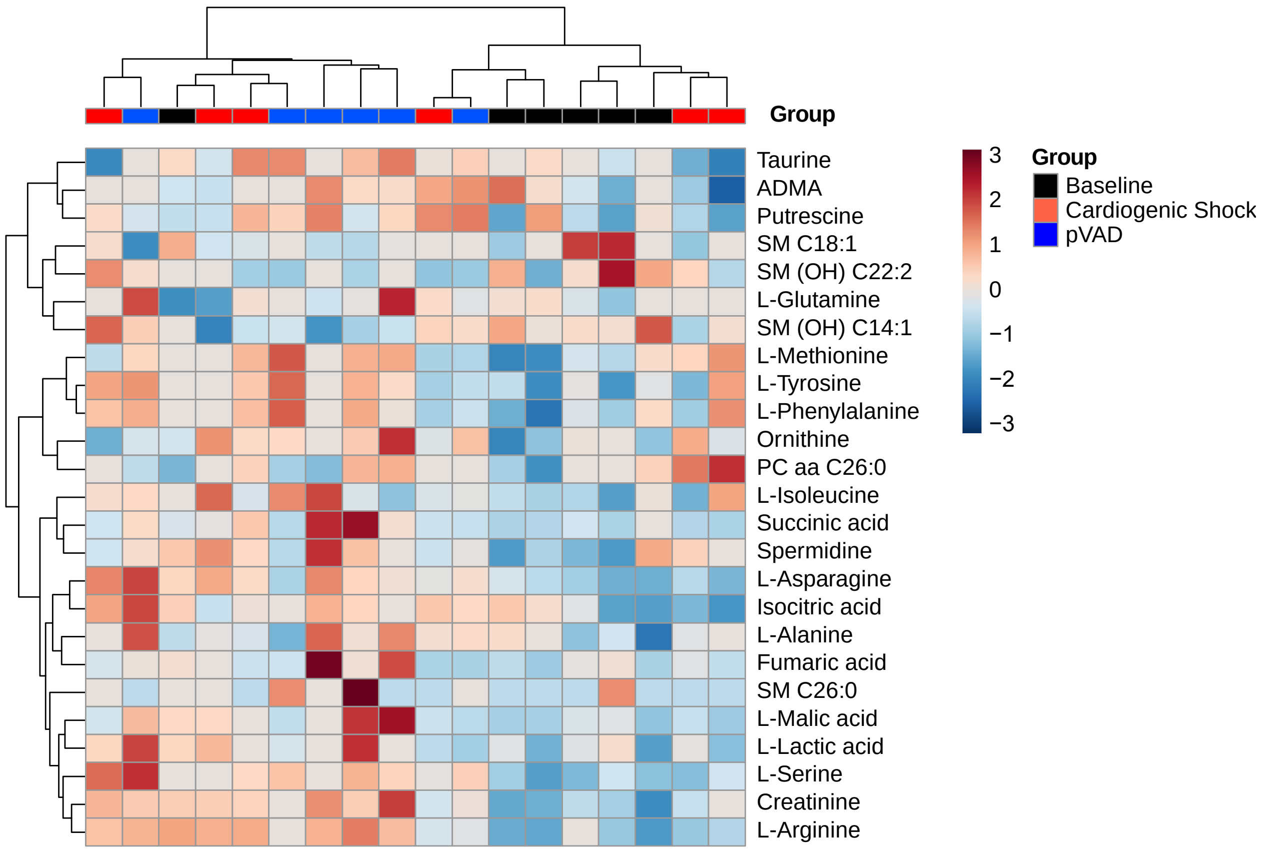

Supplementary Figure 1: *Hierarchical clustering of metabolomic analysis.*
Heatmap of metabolomic analysis corresponding to the 25 metabolites with the greatest variance across samples. Dendrograms indicate hierarchical clustering of metabolite abundance. pVAD, percutaneous ventricular assist device.

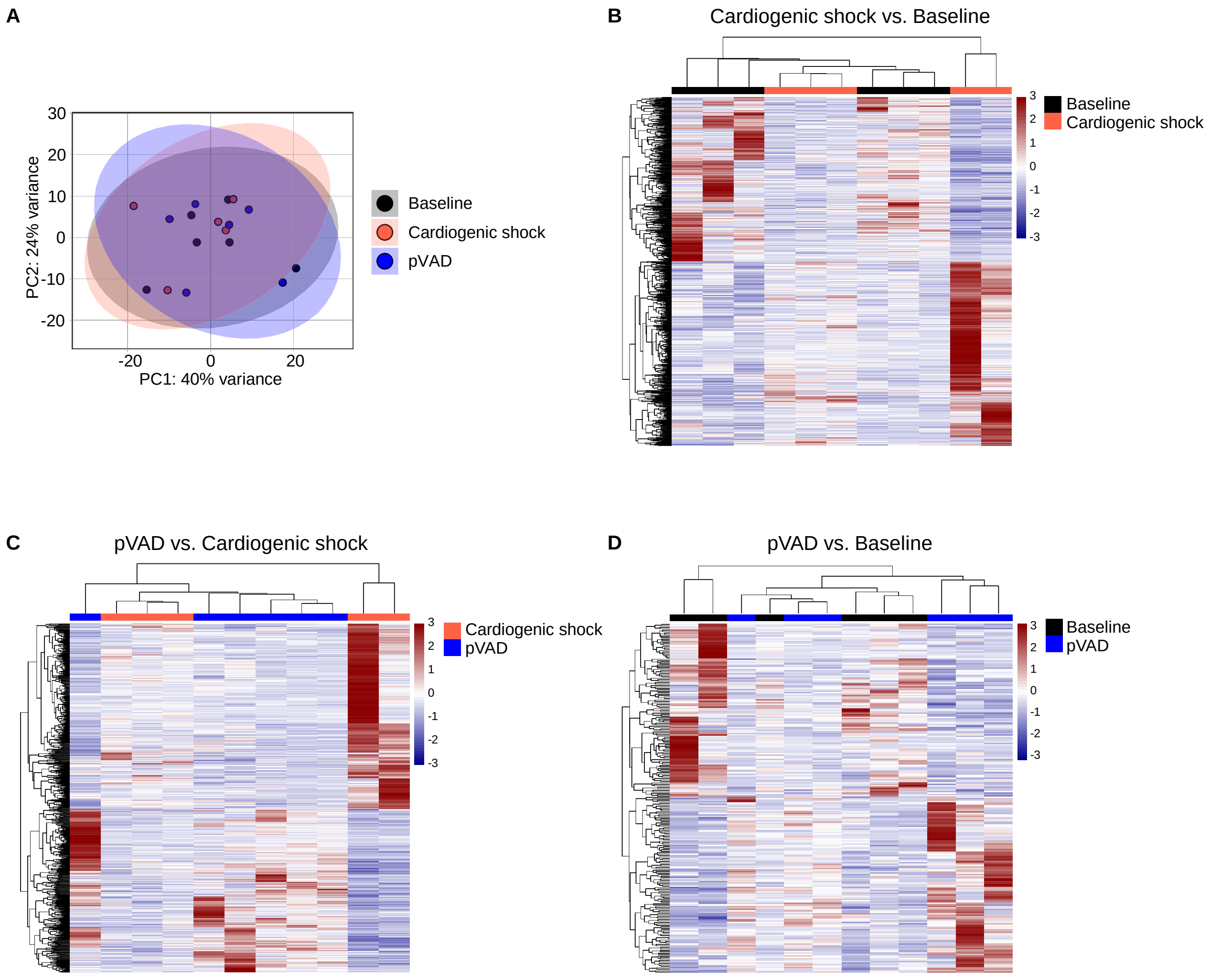

Supplementary Figure 2: *Summary of RNA sequencing results.*
(A) Principal component analysis visualizing global transcript expression. Heatmaps for the comparisons (B) Cardiogenic shock vs. Baseline, (C) pVAD vs. Cardiogenic shock, and (D) pVAD vs. Baseline. Heatmaps display differentially expressed transcripts with a |fold change| >1.5 and $p<0.05$. Dendrograms indicate hierarchical clustering of transcript expression. pVAD, percutaneous ventricular assist device.

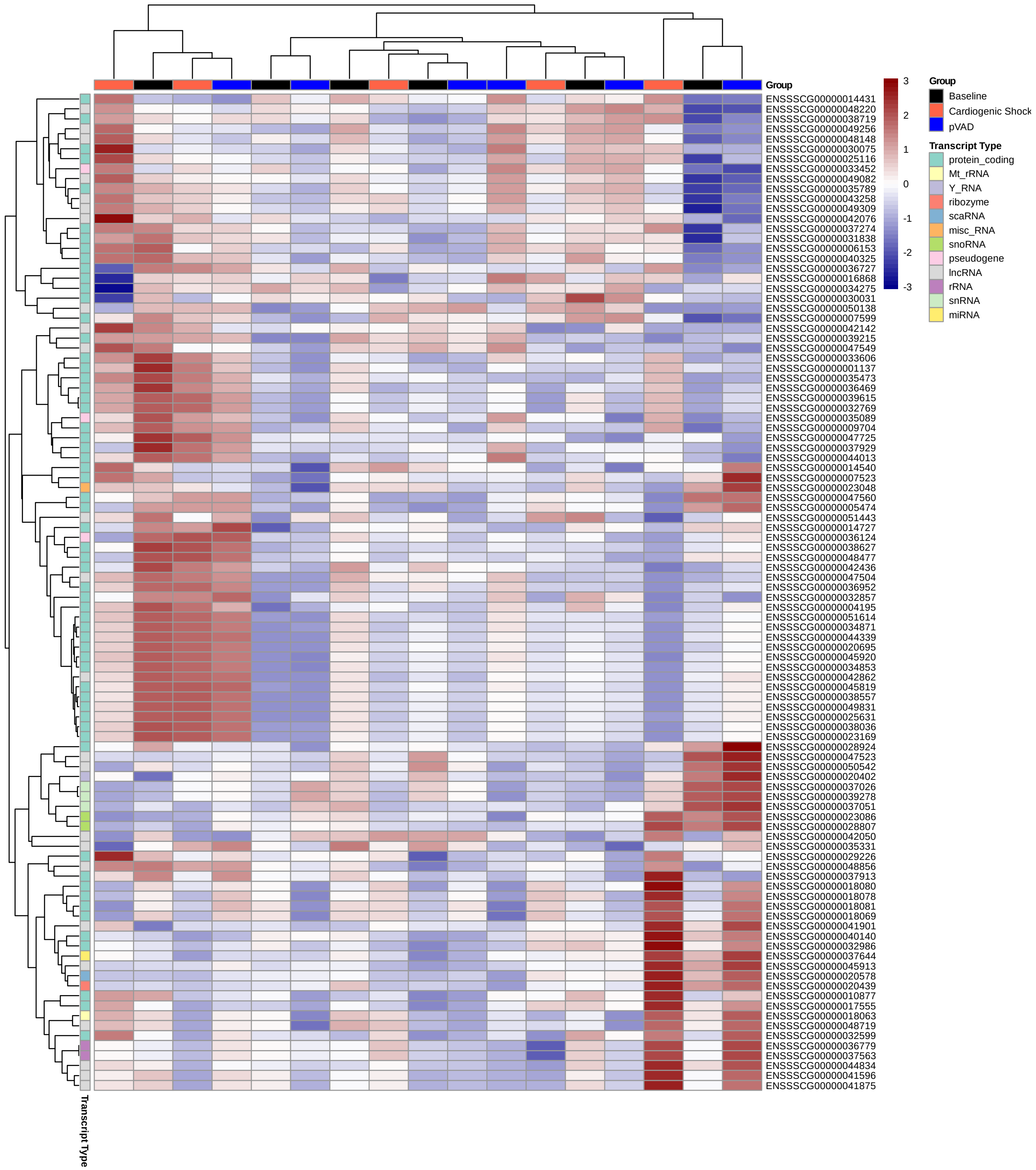

Supplementary Figure 3: *Hierarchical clustering of RNA sequencing.*

Heatmap of RNA sequencing count data corresponding to the 100 transcripts with the greatest variance across samples. Dendrograms indicate hierarchical clustering of transcript expression. pVAD, percutaneous ventricular assist device.

Supplementary Methods

Statistical analysis

Statistical analysis for the Spearman correlation between miRNA-200b expression and cardiac output was performed using GraphPad Prism 8 (GraphPad Inc., La Jolla, CA, USA). Figure 4B illustrating the 95% confidence intervals calculated by linear regression model was created using GraphPad Prism 8 (GraphPad Inc., La Jolla, CA, USA).

Metabolomic analysis

Measurement of cyclic adenosine monophosphate (cAMP) levels

Detection and quantification of cAMP levels were performed by liquid chromatography, which was coupled to tandem mass spectrometry (LC-MS/MS). Briefly, 50 µL of serum were combined with 200 µL of a mixture containing acetonitrile and methanol (50/50; [v/v]) with tenofovir as an internal standard (final concentration: 37.5 ng/mL). Samples were mixed for the duration of 30 sec total. Proteins were precipitated by incubating for 10 min at 95 °C and were then pelleted by centrifugation. The supernatant was removed and evaporated under a nitrogen stream at 40°C. Pellets were subsequently dissolved in 150 µL of water. The LC-MS/MS analysis protocol was performed as previously described (2). The HPLC-system consisted of two LC-30AD HPLC pumps, a SIL-30AC temperature controlled autosampler, a DGU-20A5 degasser, a CTO-20AC oven, and a CBM-20A control unit (Shimadzu, Duisburg, Germany), which were connected to a 5500QTRAP® mass spectrometer (Sciex, Framingham, MA, USA). The lowest concentration for the quantification of cAMP was 1.05 nM.

Measurement of TCA cycle metabolites

TCA cycle metabolites were analyzed in 50 µL of serum using an LC-MS/MS-system as previously described (3). The LC-MS/MS system was used with a setup as previously described (3). The lowest concentration for the quantification of cis-Aconitic acid was 0.052 µM, 14.5 µM for L-Lactic acid, 0.054 µM for Citric acid, 0.185 µM for Succinic acid, 0.102 µM for L-Malic acid, and 0.4 µM for Fumaric acid.

Targeted metabolomic profiling

Targeted metabolomic profiling was performed by using the AbsoluteIDQ® p180 mass spectrometry assay preparation kit (Biocrates life science AG, Innsbruck, Austria) according to the manufacturer's instructions. The LC-MS/MS system was used with a setup as described above.

Measurement of intermediates of tryptophan metabolism

Detection and quantification of intermediates of tryptophan metabolism were performed by LC-MS/MS analysis. Briefly, 50 µL of serum were combined with 200 µL of a mixture containing acetonitril and methanol (50/50; [v/v]) with caffeic acid (final concentration: 1 µM) as internal standard. The solution was mixed for 30 sec and then frozen at - 20 °C (overnight) to complete protein precipitation. After thawing, proteins were separated by centrifugation and the supernatant was evaporated under nitrogen. Next, metabolites were resolved in 150 µL of water, transferred to analysis vials, and subjected to LC-MS/MS analysis as described above. For chromatographic (GC) separation of metabolites, a C18 reversed phase column with a C18 security guard were used. The following gradient of water (solvent A) and methanol (solvent B), both containing 0.1 % (v/v) formic acid, was applied: 0-13.9 min: 5-95 % B, 14.0-14.9 min: 95 % B, 15.0-21.0 min: 5 % B. The flow rate was 0.4 mL/min at a temperature of 30°C. After chromatographic separation, metabolites were ionized in an

electrospray ionization (ESI) source. To maximize ionization yield, the ionization mode was altered between positive and negative ionization while performing the measurements. ESI parameters in positive ionization mode were as follows: electrospray voltage: 4500 V, curtain gas (CUR): 30 psi, collision gas (CAD): 9, temperature: 400°C, gas 1: 60 psi, and gas 2: 75 psi. Electrospray voltage in negative ionization mode was - 4500. All other parameters were similar compared to the positive ionization mode. Metabolites were identified by mass transitions and retention times as follows:

**Metabolite**	**Ionization mode**	**Precursor mass [Da]**	**Fragment mass [Da]**	**Retention time [min]**
L-Tryptophan	positive	205	146	5.4
L-Kynurenine	negative	207	144	3.7
2-Aminobenzoic acid	positive	138	92	7.4
3-Hydroxyanthranilic acid	negative	152	108	4.8
Hydroxykynurenine	positive	225	110	2.5

The ratio of the analytic peak area normalized to the internal standard peak area was used for quantification. Metabolite concentrations were calculated using a calibration curve, which was fitted with a quadratic regression using a weighting factor of 1/x (software: Analyst 1.7, Sciex, Framingham, MA, USA). The lowest concentration for the quantification of L-Tryptophan was 3.19 nM, 0.41 nM for L-Kynurenine, 0.4 nM for 2-Aminobenzoic acid, 0.4 nM for 3-Hydroxyanthranilic acid, and 0.15 nM for Hydroxykynurenine.

Bioinformatic analysis

Metabolomics data were analyzed with Metaboanalyst 5.0 (4). Variable Importance in Projection (VIP) was calculated by Partial Least Squares-Discriminant Analysis (PLS-DA), which is a supervised method of multivariate regression techniques. The pathway analysis was conducted based on Kyoto Encyclopedia of Genes (KEGG) metabolic pathways with the hypergeometric enrichment method. Data sets were analyzed using paired Student's t-tests. A p-value of <0.05 was considered significantly different.

<u>RNA sequencing analysis</u>

RNA sequencing

The RNA sequencing library was generated from 100 ng of total RNA using the NEBNext® Single Cell/Low Input RNA Library Prep Kit (New England BioLabs, Frankfurt, Germany) according to manufacturer's protocol. The libraries were sequenced using a NovaSeq 6000 system and the NovaSeq 6000 S2 Reagent Kit (100 cycles, paired end run) with an average of $3 * 10^7$ reads per RNA sample (Illumina, San Diego, CA, USA). A quality report for each FASTQ file was generated using the FASTQC tool (http://www.bioinformatics.babraham.ac.uk/projects/fastqc). Prior to alignment to the reference porcine genome, each sequence in the raw FASTQ files was trimmed on base call quality and sequencing adapter contamination using the Trim Galore! wrapper tool (https://www.bioinformatics.babraham.ac.uk/projects/trim_galore/). Reads shorter than 20 base pairs were removed from the FASTQ files. Trimmed reads were aligned to the reference genome using the open source short read aligner STAR (5). Feature counts were determined using the R package "Rsubread" (6). Only transcripts showing counts greater than 5 at least two times across all samples were considered for further analysis (data cleansing). Transcript annotation was performed using the R package "bioMaRt" (7).

Bioinformatic analysis

The initial study design comprised six samples for each time point, i.e. Baseline, Cardiogenic shock, and percutaneous ventricular assist device (pVAD)-mediated mechanical unloading in cardiogenic shock (Figure 1). One sample for the Cardiogenic shock time point was identified as an outlier based on total sequence number compared to the other samples. Because of these most likely technical issues that occurred during the sequencing process the sample was omitted from further analysis.

R software (version 4.0.3, R Foundation for Statistical Computing, Vienna, Austria) was used to perform downstream analysis and data visualization from the annotated raw count data. *DESeq2* (1.30.1) was employed within R to perform differential gene expression based on a negative binomial generalized linear model. First, the count data was pre-filtered to keep only rows with at least 5 reads in total. Provided sample information of the time-course study and the annotated pre-filtered raw read counts were used to create a dataset design matrix. *DESeq2* first estimates the size factor or the normalizing factor for different read depths for each gene (8). It also performs a gene-wise dispersion estimate via maximum likelihood, followed by adjustment with the empirical Bayes method. Then, *DESeq2* provides normalized count data for each contrast by negative binomial generalized linear model fitting. Three comparisons were analyzed within this dataset: changes in Cardiogenic shock compared to Baseline, changes in pVAD support in cardiogenic shock compared to Cardiogenic shock (without pVAD support), and changes in pVAD support in cardiogenic shock compared to Baseline.

Quantification and significance of differential gene expression were determined using the Wald test that provides quantile-normalized read counts together with $\log_2$ fold change for each contrast, p-value, and adjusted p-value corrected for multiple testing using the Benjamini-Hochberg procedure. All contrasts were compared with one another to identify differentially expressed transcripts and to analyze whether they are specific or overlapping with other groups and are changing in the same or opposite direction. Final read count data was transformed using regularized logarithm for visualization purposes.

Unsupervised Principal Component Analysis (PCA) plot was generated by using the *plotPCA* function within *DESeq2*. *ggplot2* (3.3.3) was employed to generate volcano plots and bar plots. Heatmap visualization and hierarchical clustering were performed with Ward’s minimum squared variance algorithm, and dendrograms were generated by Euclidean distance via pheatmap (1.0.12). The *enrichR* (3.0) R interface package was used to perform and import pathway enrichment analysis data from the web-based tool *Enrichr* (9). Generation and analysis of Venn diagrams were performed with the software *Vennplex* (10). RNA-seq data have been deposited to the GEO database under the accession number GSE199090.

References

1. Udesen NLJ, Helgestad OKL, Banke ABS, Frederiksen PH, Josiassen J, Jensen LO, et al. Impact of Concomitant Vasoactive Treatment and Mechanical Left Ventricular Unloading in a Porcine Model of Profound Cardiogenic Shock. *Crit Care* (2020) 24(1):95. doi: 10.1186/s13054-020-2816-8.
2. Beste KY, Burhenne H, Kaever V, Stasch JP, Seifert R. Nucleotidyl Cyclase Activity of Soluble Guanylyl Cyclase Alpha1beta1. *Biochemistry* (2012) 51(1):194-204. Epub 2011/11/30. doi: 10.1021/bi201259y.
3. Winterhoff M, Chen F, Sahini N, Ebensen T, Kuhn M, Kaever V, et al. Establishment, Validation, and Initial Application of a Sensitive Lc-Ms/Ms Assay for Quantification of the Naturally Occurring Isomers Itaconate, Mesaconate, and Citraconate. *Metabolites* (2021) 11(5). Epub 2021/05/01. doi: 10.3390/metabo11050270.
4. Pang Z, Chong J, Zhou G, de Lima Morais DA, Chang L, Barrette M, et al. Metaboanalyst 5.0: Narrowing the Gap between Raw Spectra and Functional Insights. *Nucleic Acids Res* (2021) 49(W1):W388-W96. Epub 2021/05/22. doi: 10.1093/nar/gkab382.
5. Dobin A, Davis CA, Schlesinger F, Drenkow J, Zaleski C, Jha S, et al. Star: Ultrafast Universal Rna-Seq Aligner. *Bioinformatics* (2013) 29(1):15-21. Epub 2012/10/30. doi: 10.1093/bioinformatics/bts635.
6. Liao Y, Smyth GK, Shi W. Featurecounts: An Efficient General Purpose Program for Assigning Sequence Reads to Genomic Features. *Bioinformatics* (2014) 30(7):923-30. Epub 2013/11/15. doi: 10.1093/bioinformatics/btt656.
7. Durinck S, Moreau Y, Kasprzyk A, Davis S, De Moor B, Brazma A, et al. Biomart and Bioconductor: A Powerful Link between Biological Databases and Microarray Data Analysis. *Bioinformatics* (2005) 21(16):3439-40. Epub 2005/08/06. doi: 10.1093/bioinformatics/bti525.
8. Love MI, Huber W, Anders S. Moderated Estimation of Fold Change and Dispersion for Rna-Seq Data with Deseq2. *Genome Biol* (2014) 15(12):550. Epub 2014/12/18. doi: 10.1186/s13059-014-0550-8.
9. Chen EY, Tan CM, Kou Y, Duan Q, Wang Z, Meirelles GV, et al. Enrichr: Interactive and Collaborative Html5 Gene List Enrichment Analysis Tool. *BMC Bioinformatics* (2013) 14:128. Epub 2013/04/17. doi: 10.1186/1471-2105-14-128.
10. Cai H, Chen H, Yi T, Daimon CM, Boyle JP, Peers C, et al. Vennplex--a Novel Venn Diagram Program for Comparing and Visualizing Datasets with Differentially Regulated Datapoints. *PLoS One* (2013) 8(1):e53388. Epub 2013/01/12. doi: 10.1371/journal.pone.0053388.
